# Nuclear IKKα mediates microRNA-7/-103/107/21 inductions to downregulate maspin expression in response to HBx overexpression

**DOI:** 10.18632/oncotarget.10462

**Published:** 2016-07-07

**Authors:** Wen-Shu Chen, Liang-Chih Liu, Chia-Jui Yen, Yun-Ju Chen, Jhen-Yu Chen, Chien-Yi Ho, Shu-Hui Liu, Ching-Chow Chen, Wei-Chien Huang

**Affiliations:** ^1^ Department of Pharmacology, National Taiwan University, Taipei, Taiwan; ^2^ Center for Molecular Medicine, China Medical University Hospital, Taichung, Taiwan; ^3^ Division of Breast Surgery, Department of Surgery, China Medical University Hospital, Taichung, Taiwan; ^4^ Department of Family Medicine, China Medical University Hospital, Taichung, Taiwan; ^5^ Graduate Institute of Cancer Biology, China Medical University, Taichung, Taiwan; ^6^ School of Medicine, China Medical University, Taichung, Taiwan; ^7^ The Ph.D. Program for Cancer Biology and Drug Discovery, China Medical University, Taichung, Taiwan; ^8^ Internal Medicine, National Cheng-Kung University, Tainan, Taiwan; ^9^ Department of Medical Research, E-DA Hospital, Kaohsiung, Taiwan; ^10^ Department of Biological Science & Technology, I-Shou University, Kaohsiung, Taiwan; ^11^ Department of Health Care and Social Work, Yu Da University of Science and Technology, Miaoli, Taiwan; ^12^ Department of Biotechnology, Asia University, Taichung, Taiwan

**Keywords:** IKKα, maspin, HBx, microRNA, hepatocellular carcinoma

## Abstract

Maspin is a tumor suppressor that stimulates apoptosis and inhibits metastasis in various cancer types, including hepatocellular carcinoma (HCC). Our previous study has demonstrated that HBx induced microRNA-7, 103, 107, and 21 expressions to suppress maspin expression, leading to metastasis, chemoresistance, and poor prognosis in HCC patients. However, it remains unclear how HBx elicits these microRNA expressions. HBx has been known to induce aberrant activation and nuclear translocation of inhibitor-κB kinase-α (IKKα) to promote HCC progression. In this study, our data further revealed that nuclear IKKα expression was inversely correlated with maspin expression in HBV-associated patients. Nuclear IKKα but not IKKβ reduced maspin protein and mRNA expression, and inhibition of IKKα reverses HBx-mediated maspin downregulation and chemoresistance. In response to HBx overexpression, nuclear IKKα was further demonstrated to induce the gene expressions of microRNA-7, −103, −107, and −21 by directly targeting their promoters, thereby leading to maspin downregulation. These findings indicated nuclear IKKα as a critical regulator for HBx-mediated microRNA induction and maspin suppression, and suggest IKKα as a promising target to improve the therapeutic outcome of HCC patients.

## INTRODUCTION

Hepatocellular carcinoma (HCC), a leading cause of cancer-related death worldwide, shows high metastasis and recurrence rates and chemoresistance [1, 2]. Risk factors for HCC include aflatoxin, cirrhosis and hepatitis, chronic hepatitis B virus (HBV) and hepatitis C virus (HCV) infections, alcoholic liver diseases, and nonalcoholic fatty liver diseases [3]. Among these risk factors, chronic inflammation induced by HBV or HCV accounts for the majority of liver cancer development [4, 5]. The causal roles of HBV and HCV infections in HCC tumorigenesis have been demonstrated as the efficient reduction of HCC development by eradicating these viruses [6]. However, distinct pathophysiologic mechanisms underlying HBV- and HCV-related hepatocarcinogenesis have been proposed [7].

Hepatitis B virus X protein (HBx), a critical antigen involved in HBV-associated liver diseases, determines an unique profile of gene expression in the host liver cells and contributes to HCC formation. As a multifunctional regulator, HBx modulates several cellular processes such as oxidative stress, DNA repair, signal transduction pathways, transcriptional regulations, protein degradation, cell cycle progression, apoptosis, and genetic stability by direct or indirect interaction with host factors [8, 9]. Several reports have demonstrated that HBx promotes HCC cell proliferation by downregulation of p16 protein expression and upregulation of cyclin D1 protein expression via activation of the MEK/ERK and PI3K/Akt signaling pathways [10–13]. HBx also promotes tumor metastasis by inducing cyclooxygenase-2 and matrix metalloproteinase-1, 2, 3, and 9 expressions, and repressing E-cadherin and fibronectin type III domain containing 3B (FNDC3B) expressions [1, 14–18]. Moreover, downregulation of p53 and p21, inhibition of caspase-3 activation, and upregulation of anti-apoptotic protein survivin and multi-drug resistance proteins were reported to mediated the chemoresistance of HBx-expressing hepatocellular carcinoma cells [19–22]. Disruption of HBx protein expression can effectively inhibit tumor growth and enhance chemotherapy-induced apoptosis in hepatocellular carcinoma cells [23–25]. Notably, microRNAs have been recognized as important regulators in HBV-related HCC progression via targeting gene expressions [26, 27].

Recently, our study demonstrated that maspin, a mammary serine protease inhibitor, was specifically reduced by HBx protein in HBV-associated HCC patients, [28]. The downregulation of maspin promoted cell motility and rendered resistance to anoikis and chemotherapy in HCC cells. Furthermore, the increased microRNA-7, −103, −107, and −21 in response to HBx overexpression was demonstrated to directly target maspin mRNA, and the levels of microRNA-7/21/107 were correlated to poor prognosis in HBV-associated HCC patients. However, the molecular mechanisms underlying these HBx-induced microRNA expressions remain to be elucidated.

Nuclear factor κB (NF-κB) pathway controls many important pathological processes including inflammation, immunity, cell proliferation, differentiation, survival, as well as cancer progression [29]. Aberrant NF-κB activation in response to various stimuli can promote cancer invasion, metastasis, and chemoresistance in liver cancer [30, 31]. Activation of NF-κB signaling pathway by HBx has been well documented [8, 32]. In addition, our previous study also demonstrated that HBx induced the nuclear localization of IKKα, a upstream kinase for NF-κB activation, in an Akt phosphorylation-dependent manner to promote the migration and invasion of HCC cells via phosphorylating histone H3 at Ser^10^ [33]. Phosphorylation of Histone H3 Ser^10^ on *maspin* promoter by RANKL-activated nuclear IKKα was proposed to directly repress *maspin* transcription through subsequent DNA methylation [34]. However, this histone posttranslational modification was widely reported to enhance transcription of most genes involved in chromosome decondensation and cell-cycle progression during mitosis and meiosis as well as the NF-κB-targeted gene expressions during inflammation [35–37]. The closed proximity to other modifiable residues on the histone H3 tail leads to the cross-talk of serine 10 phosphorylation with the transcription-activating acetylation at lysine 9 and lysine 14 [38]. Thus, maspin suppression by nuclear IKKα may involve an indirect regulation through inducing gene expression of intermediate suppressors such as microRNAs rather than DNA methylation merely.

In the present study, we found an inverse correlation between phosphorylated nuclear IKKα and maspin protein expression in HBV-associated HCC patients. The activity and nuclear translocation of IKKα but not IKKβ was crucial for HBx-mediated maspin downregulation and chemoresistance in HCC cells. Furthermore, nuclear IKKα-induced microRNA-7, −21, −103, and −107 expressions relying on histone H3 Ser10 phosphorylation to disrupt maspin mRNA stability and translation. These results provide new insights into the molecular mechanisms of maspin suppression in response to HBx, and revealed nuclear IKKα as a prognostic biomarker and a potential therapeutic target to improve the clinical outcome of HBV-associated HCC patients.

## RESULTS

### Nuclear IKKα significantly correlates with low levels of maspin expression in HBV-associated HCC patients

**Figure 1:**
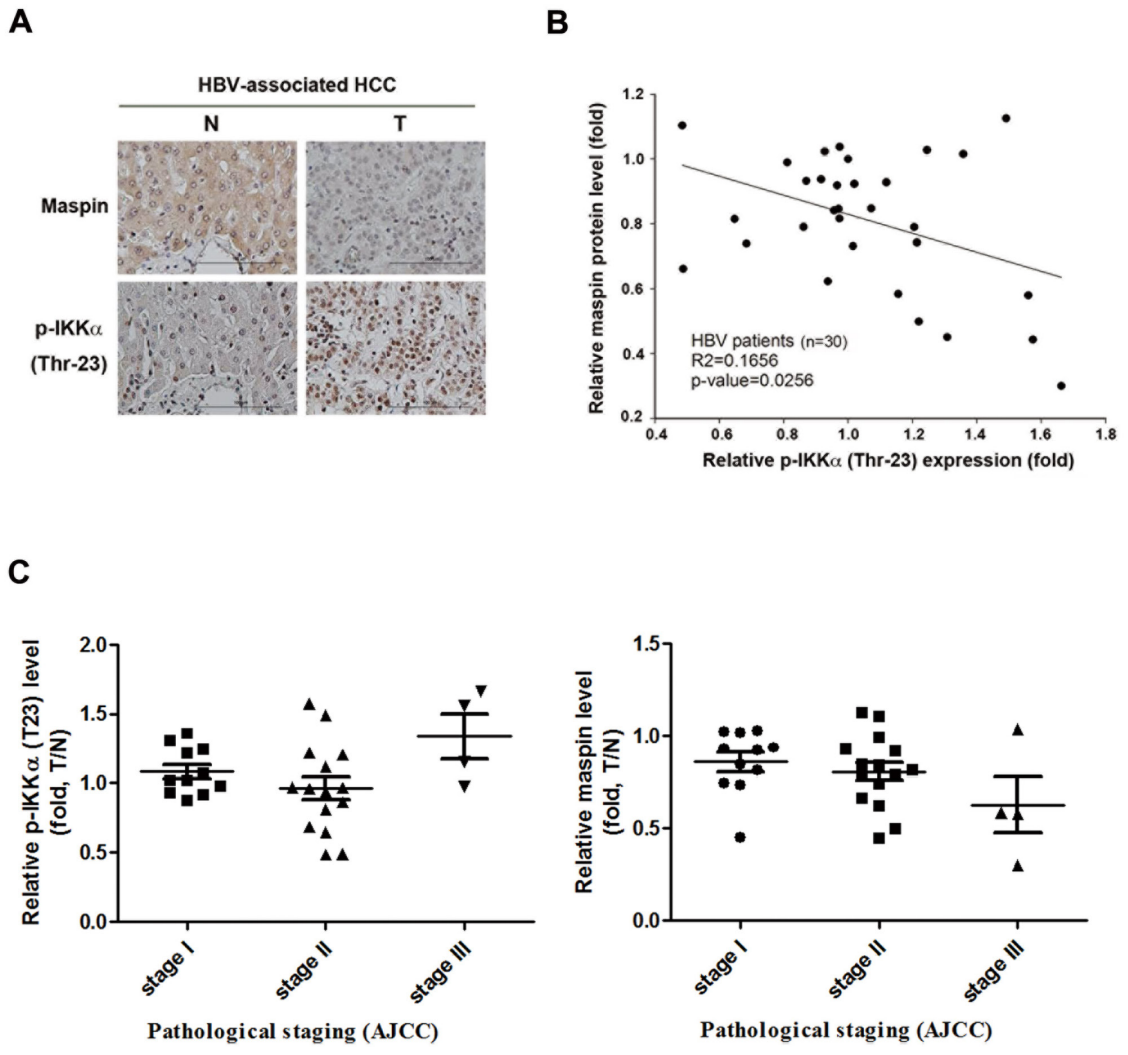
Inverse correlation between phospho-IKKα and maspin expression in HBV-associated HCC patients **A.** Representative immunohistochemical staining of maspin (top) and phospho-IKKα (Thr-23) (bottom) in HBV-associated HCC tumor liver tissues (T) and adjacent normal liver tissues (N) was shown. Scale bar: 100 μm. **B.** and **C.** Total lysates from HBV-associated HCC tumor liver tissues were prepared and subjected to Western blot with anti- phospho-IKKα (Thr-23), maspin, and ERK antibodies. The coefficient of determination (r^2^) between IKK phosphorylation and maspin expression levels was analyzed by simple regression with normalization to ERK protein level (n=30). The clinical association of p-IKKα and maspin levels with the stages of HBV-associated HCC was further analyzed by a Student's t-test.

Our previous study has demonstrated that HBx-mediated maspin suppression contributed to HBV-induced HCC progression [28]. We also demonstrated that HBx induced nuclear IKKα translocation through Akt-dependent Thr-23 phosphorylation to promote motility of hepatocarcinoma cells [33]. Furthermore, cytokine-activated nuclear IKKα has been reported to repress maspin to promote metastasis of prostate cancer [34]. Therefore, the correlation between nuclear IKKα and maspin suppression in HBV-associated HCC tumors was first examined. The phosphorylation of IKKα at Thr-23, which was recognized as a marker for nuclear localization, was elevated and predominantly localized in the nucleus, and was inversely correlated with maspin expression in HBV-associated HCC tumors (Figure 1A and 1B, respectively), supporting the involvement of nuclear IKKα in maspin suppression. Additionally, the clinical association of IKKα T23 phosphorylation and maspin expression with the status of HBV-associated HCC tumors was also analyzed. In the comparison to the normal tissues, IKKα T23 phosphorylation is up-regulated and maspin expression is downregulated in the stage III but not in stage I and II HCC tumor tissues (Figure 1C).

### Nuclear IKKα but not IKKβ mediated HBx-dependent maspin suppression and chemoresistance in HCC cells

**Figure 2:**
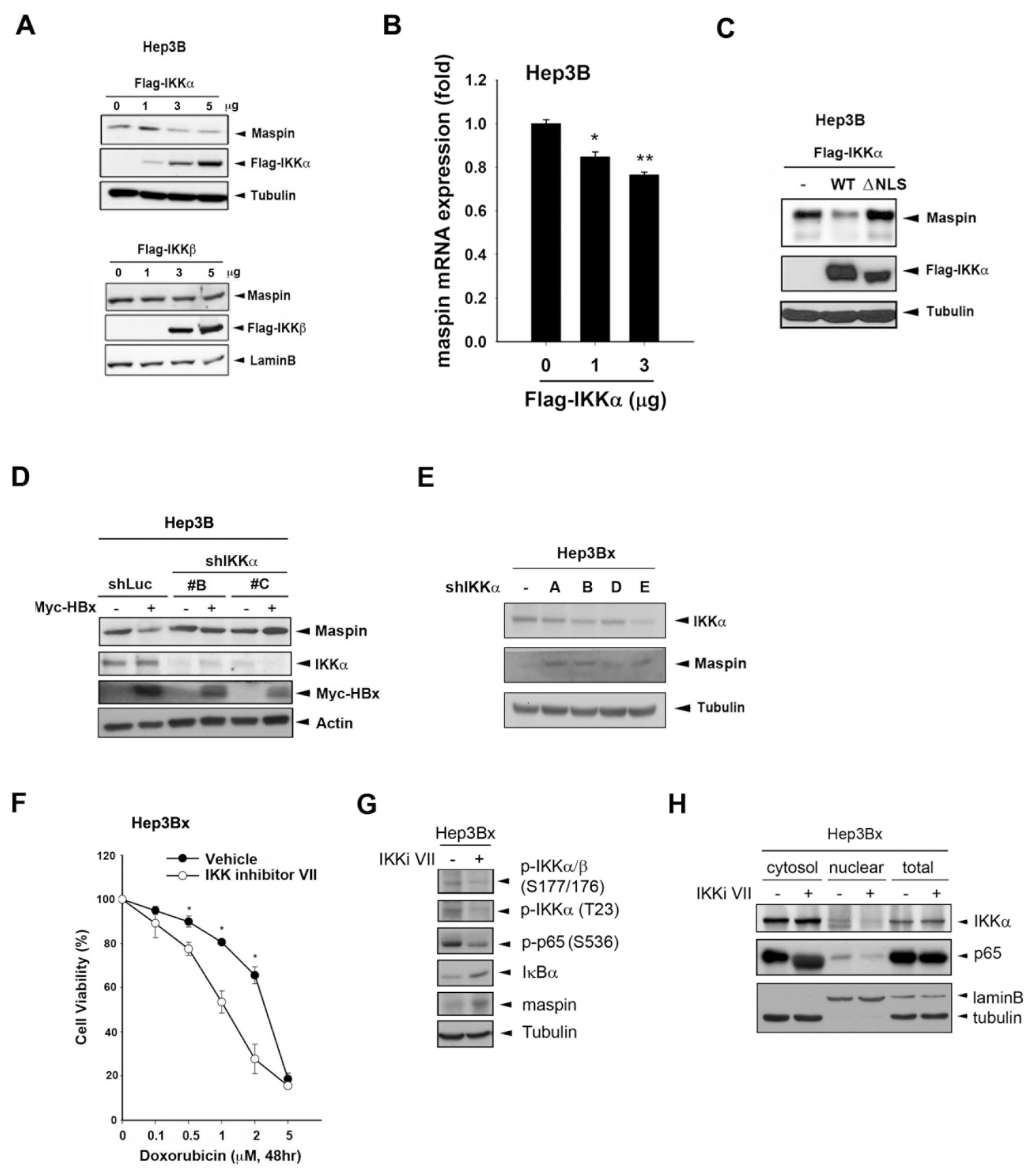
Nuclear IKKα mediated HBx-dependent maspin suppression and chemoresistance **A-D.** Total lysates from Hep3B cells transfected with IKKα and its NLS deletion mutant IKKβ, myc-HBx along with IKKα shRNA were subjected to Western blot analysis with indicated antibodies. **E.** Hep3Bx cells were transfected with IKKα shRNA for 3 days, and then total lysates were prepared and subjected to Western blot analysis. **F.** Hep3Bx cells treated with doxorubicin in the presence or absence of IKK inhibitor VII for 24 hours were subjected to MTT assays to determine the cell viability (n=3). **G.** and **H.** After treatment with IKKα inhibitor VII for 48 hours, total, nuclear, and cytosolic lysates from Hep3Bx cells were prepared and subjected to Western blot analysis. The difference was calculated by a Student's t-test (*: p<0.05; **: p<0.01)

Since the IKK-NF-κB signaling pathway plays an important role in the development of HCC, the regulatory function of IKKα and IKKβ, the essential kinases controlling canonical and noncanonical NF-κB signaling, in maspin expression were further examined. Overexpression of IKKα but not IKKβ downregulated maspin protein expression as well as the mRNA level in Hep3B cells (Figure 2A and 2B). Furthermore, the maspin suppression was abolished by mutation of the IKKα nuclear localization signal (NLS) (Figure 2C). Our previous study has demonstrated that HBx suppressed maspin expression and enhanced chemoresistance [28]. The role of IKKα in HBx-mediated maspin suppression was further examined by silencing of IKKα with shRNA. Indeed, knockdown of IKKα prevented HBx-induced maspin suppression in transient (Figure 2D) and stable (Figure 2E) HBx transfectants of Hep3B cells. To further verify the critical role of IKKα in HBx-mediated chemoresistance, IKK inhibitor VII was utilized and the cytotoxicity of Hep3Bx cells was determined by MTT assay. We found that IKK inhibitor VII significantly increased the sensitivity of Hep3Bx cells to doxorubicin (Figure 2F). Additionally, the effects of IKK inhibitor VII on IKKα activity and nuclear translocation as well as maspin expression were also addressed. As shown in Figure 2G, the expression of, phospho-IKKα/β (Ser177/176), phospho-IKKα (Thr-23), and phospho-p65 (Ser536) were downregulated whereas the expression of IκBα was upregulated by IKK inhibitor VII. Accompanied with the IKKα activity inhibition, the nuclear translocation of IKKα was decreased (Figure 2H) and the expression of maspin was significantly restored in Hep3Bx cells (Figure 2G). These data support the essential role of nuclear IKKα in HBx-induced maspin downregulation and chemoresistance.

### Nuclear IKKα downregulats maspin expression through disrupting its mRNA stability

**Figure 3:**
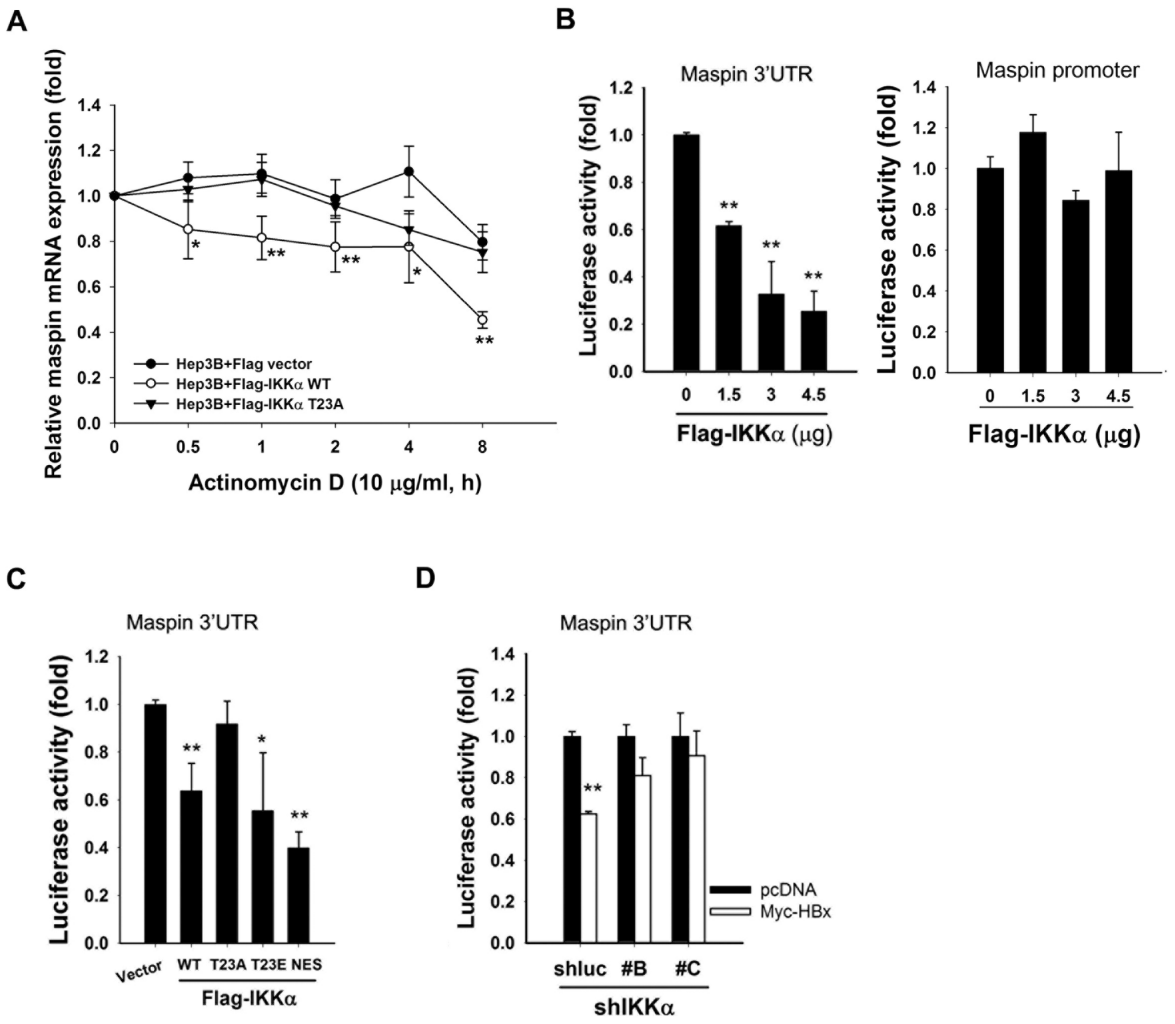
Nuclear IKKα disrupted maspin mRNA stability to mediate HBx-dependent maspin suppression **A.** Hep3B cells were transfected with Flag-IKKα wild-type and T23A mutant for 3 days and then harvested total RNA at the indicated time points after actinomycin D (10 μ0g/ml) treatment to examine the stability of maspin mRNA by RT-qPCR (n=5). **B-D.** HEK-293 cells were transfected with maspin 3′UTR or promoter-luciferase gene, Flag-IKKα WT and its mutants, or IKKα shRNA with myc-HBx expression for 24 hours and then were subjected to luciferase activity assays (n=3). The difference was calculated by a Student's t-test (*: p<0.05; **: p<0.01).

Although cytokine-activated nuclear IKKα has been suggested to suppress maspin transcription in prostate cancer cells [34], Overexpression of IKKα wild-type but not its T23A mutant significantly decreased maspin mRNA stability in the presence of actinomycin D (Figure 3A), indicating that a post-transcriptional regulation might be involved in nuclear IKKα-mediated maspin suppression. To further address whether nuclear IKKα mediates maspin suppression via affecting maspin-3′UTR activity, IKKα and maspin-3′UTR luciferase were co-transfected into HEK-293 cells. Overexpression of IKKα suppressed the luciferase activity dose-dependently (Figure 3B, left). However, there was no suppressive effect of IKKα on maspin promoter activity, suggesting that the post-transcriptional regulation is more critical for nuclear IKKα-mediated maspin suppression (Figure 3B, right). Furthermore, IKKα T23A mutation, but not the nuclear-prone IKKα T23E and IKKα NES mutations, abolished IKKα-mediated maspin 3′UTR suppression (Figure 3C). Additionally, silence of IKKα reversed HBx-mediated suppression of maspin 3′UTR activity, which was found to be mediated by microRNAs induction in our previous study (Figure 3D), suggesting the suppression of maspin mRNA stability by nuclear IKKα through induction of microRNAs.

### Nuclear IKKα induces miR-7, −103, −107, and −21 to mediate HBx-dependent maspin suppression

**Figure 4:**
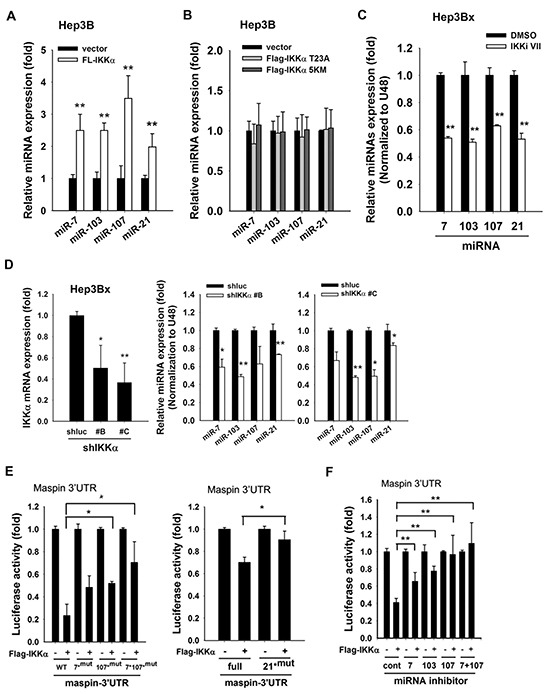
IKKα is involved in HBx-induced maspin suppression and maspin-targeting miRNA expressions **A.** and **B.** Total RNA extracted from Hep3B cells transfected with or without Flag-IKKα or mutants (T23A and 5KM) were subjected to RT-qPCR to examine the expression levels of indicated microRNAs (n=3). **C.** and **D.** The total RNA extracted from IKKαi VII-treated or IKKα-knockdown Hep3Bx cells were analyzed by RT-qPCR. The expression levels of IKKα and maspin-targeting miRNAs were normalized to GAPDH and U48, respectively (n=3). **E.** HEK-293 cells were transfected with maspin 3′UTR-luciferase gene or its mutants, and Flag-IKKα for 24 hours and then were subjected to luciferase activity assays (n = 3). **F.** HEK-293 cells were co-transfected with maspin 3′UTR-luciferase gene, Flag-tagged IKKα, and miRNA inhibitors for 48 hours and then were subjected to luciferase activity assays (n = 4). The difference was calculated by a Student's t-test (*: p<0.05; **: p<0.01).

Our previous study has demonstrated that microRNA-7/21/103/107 mediated HBx-induced maspin suppression. To further verify the involvement of these microRNAs in nuclear IKKα-mediated maspin downregulation, the expression levels of these microRNAs in response to IKKα overexpression and IKKα inhibition were analyzed. Indeed, overexpression of wild-type IKKα (Figures 4A) but not its T23A nor NLS deletion mutants (Figure 4B) increased the expressions of miR-7, −103, −107, and −21 in Hep3B cells. Moreover, inhibition of IKKα by IKK inhibitor VII and by different IKKα shRNAs significantly decreased these miRNA expressions in Hep3Bx cells (Figures 4C and 4D, respectively). Furthermore, the inhibitory effect of IKKα on maspin 3′UTR-luciferase activity was attenuated by individual or combinatory mutations on maspin 3′UTR targeted sites for miR-7, miR-107, or miR-21 (Figure 4E). The inhibition of maspin 3′UTR activity by IKKα was also partially reversed by the inhibitors against miR-7, −103, or −107 alone and was greatly reversed by the combination of miR-7 and −107 inhibitors (Figure 4F). These data suggested that nuclear IKKα can upregulate miR-7, −103, −107, and −21 expressions to suppress maspin expression in Hep3Bx cells.

### Nuclear IKKα coordinates the transcriptional activity of NF-κB to mediate microRNA-7/21/103/107 expressions in HBx-expressing HCC cells

**Figure 5:**
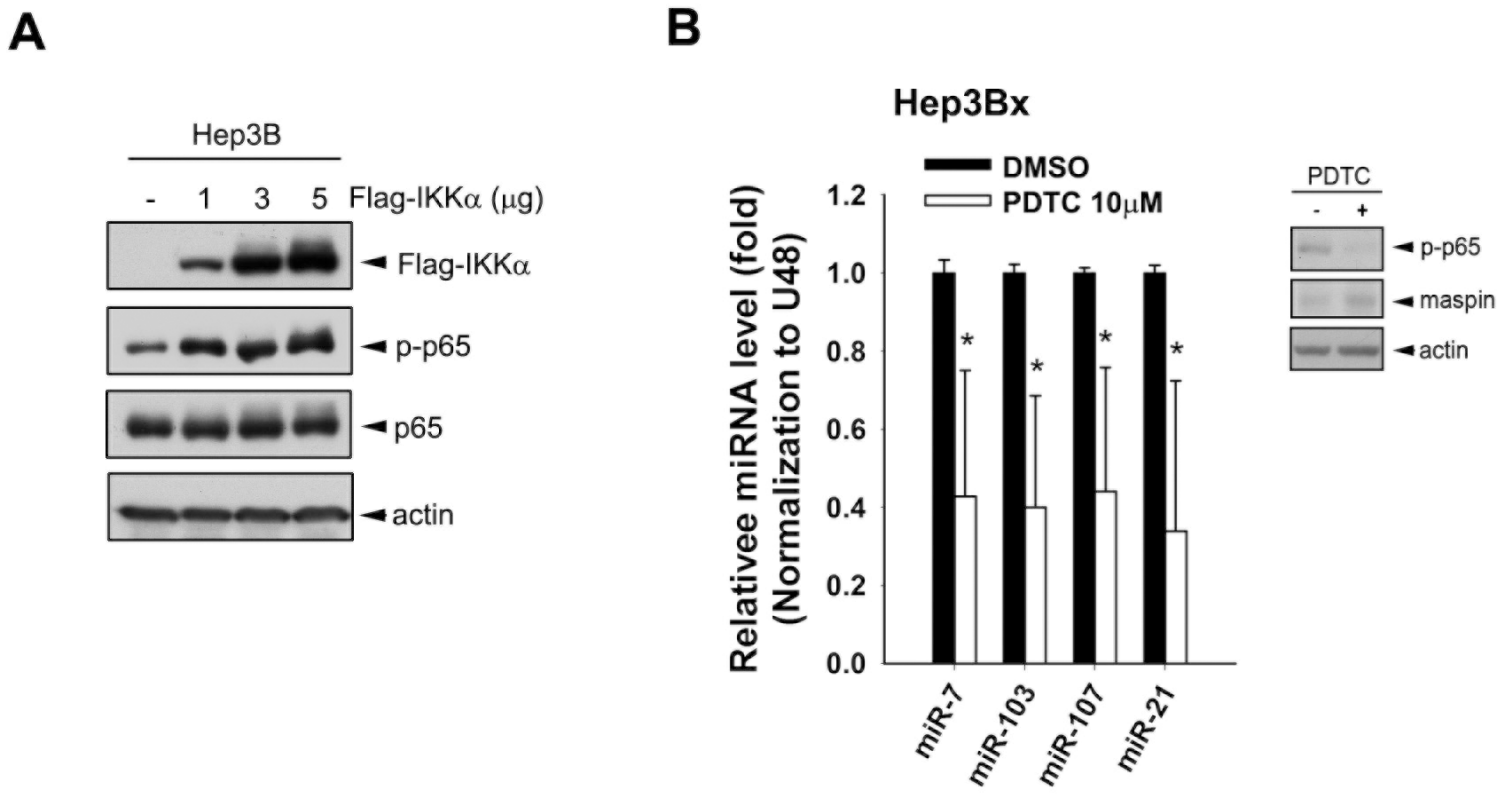
NF-κB activated by IKKα overexpression was involved in HBx-induced miRNA expressions **A.** Total lysates prepared from Hep3B cells transfected with increasing amount of IKKα were subjected to Western blot analysis with indicated antibodies. **B.** The total RNA and protein lysates extracted from Hep3Bx cells treated with NFκB inhibitor PDTC, were analyzed by RT-qPCR and Western blot analysis. The expression levels of maspin-targeting miRNAs were normalized to U48 (n=4).

**Figure 6:**
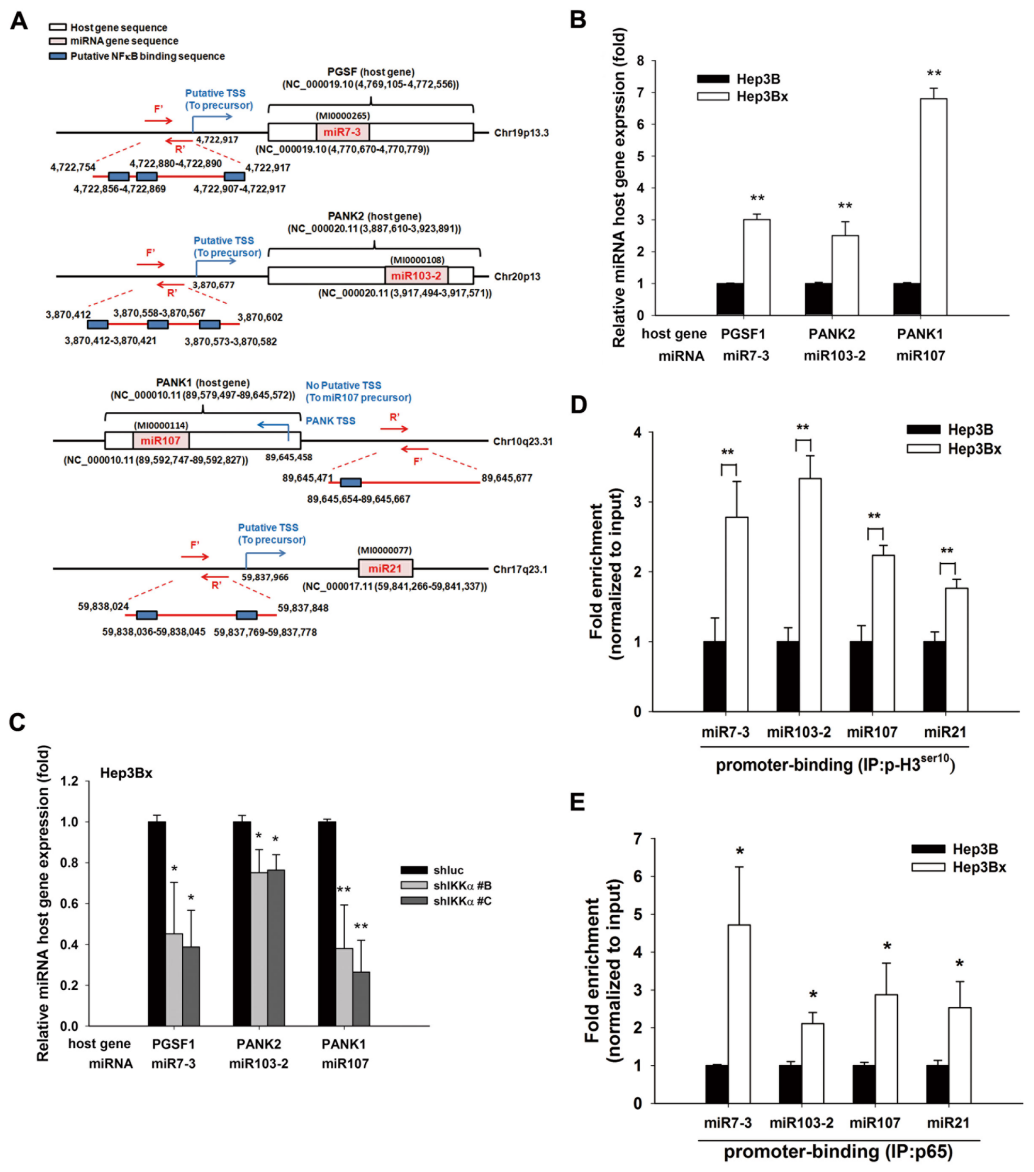
IKKα-activated NF-κB pathway transcriptional regulated HBx-mediated miRNAs and their host gene expression **A.** The diagram illustrates the gene locations of miRNA-7, −103, −107, and −21 and their predicted promoter regions. **B.** and **C.** The total RNA extracted from Hep3B, Hep3Bx, and IKKα-knockdown Hep3Bx cells was analyzed by RT-qPCR. The expression levels of miRNA host genes were normalized to GAPDH (n=4). **D.** and **E.** Total lysate from Hep3B and Hep3Bx cells were subjected to ChIP assays with anti-H3 phospho-Ser10 and anti-p65 antibodies, followed by RT-qPCR with specific primers for indicated gene promoters. The difference was calculated by a Student's t-test. *p<0.05; **p<0.01.

Our previous study has demonstrated that nuclear IKKα enhanced NFκB-mediated gene transcription by tipping CBP binding preference [39]. Overexpression of IKKα increased the active phosphorylation of p65 in Hep3B cells, and NFκB inhibitor PDTC downregulated miR-7, −103, −107, and −21, thereby restoring maspin expression in Hep3Bx cells (Figure 5A and 5B, respectively). These results suggest that HBx transcriptionally induced these microRNAs in a nuclear IKKα/NFκB-dependent manner to suppress maspin expression. The putative binding element for NF-κB is commonly observed on the promoter regions of these microRNAs’ host genes. The upstream regions and putative transcription starting site (TSS) of these microRNA precursors genes were predicted by using three independent database, TRANSFAC [40], JASPAR [41], and TFBIND [42] and were illustrated in Figure 6A, suggesting that HBx could elevate these microRNAs via epigenetically upregulating their host genes in a nuclear IKKα-dependent manner. Indeed, the gene expressions of their respective host genes including pituitary gland specific factor 1 (PGSF1) for miR-7, pantothenate kinase 2 (PANK2) for miR-103, and pantothenate kinase 1 (PANK1) for miR-107 were also elevated in Hep3Bx cells but suppressed by the silence of IKKα (Figure 6B and 6C). Moreover, accumulating evidence has indicated that the phosphorylation of histone H3 at serine 10 is necessary and sufficient for transcriptional activation, and IKKα/NFκB signaling is one of the cell-signaling cascades leading to this event [38, 43]. Therefore, we performed chromatin immunoprecipitation (ChIP) assays to compare the level of phospho-H3 Ser^10^ on the promoters of these microRNAs in Hep3B and Hep3Bx cells. Consistently, the level of phospho-H3 Ser^10^ on the promoters of these microRNAs were much higher in Hep3Bx cells than Hep3B cells, indicating the chromatin fiber were more accessible to facilitate the binding of transcription factors in Hep3Bx cells (Figure 6D). To further demonstrate NFκB is the major transcription factor participated in the transcription of these miRNAs, the promoter binding ability of NFκB was further examined in Hep3B and Hep3Bx cells. As shown in Figure 6E, the binding ability of p65 on these miRNA promoters was significantly enhanced in Hep3Bx cells than Hep3B cells, strengthening the crucial role of NFκB in HBx-induced transcription of these miRNAs. These data indicated that nuclear IKKα might be responsible for phosphorylating histone H3 at Ser10 to facilitate the expressions of these NF-κB-dependent microRNA, resulting in maspin downregulation.

## DISCUSSION

It is well-accepted that constitutive NF-κB signaling activation promotes cancer development by increasing cell proliferation, angiogenesis, metastasis, and resistance to apoptotic stimuli [44]. It has been known that activation of NF-κB signal by both chronic hepatitis virus HBV and HCV infections contributes to the development of HCC [45]. The IKKα kinase complex is the master regulator for NF-κB activation [46, 47]. High expression levels of IKKα and IKKβ have found to be necessary for the malignant properties of liver cancer [30]. However, the role of IKKs on virus infection-mediate gene regulation and tumor progression need to be further investigated. Previously, we demonstrated that HBx activates Akt to phosphorylate IKKα at T23 residue, thus promoting IKKα nuclear localization. Furthermore, we also revealed the unique role of HBx protein in maspin suppression through microRNA induction to promote HBV-associated HCC tumor progression [28]. In the present study, the mechanism underlying HBx-dependent microRNA inductions was further uncovered. Nuclear activated IKKα transcriptionally up-regulates microRNA-7, −21, −103, and −107 expressions to target maspin 3′UTR, contributing to HBx-mediated maspin suppression and subsequent chemoresistance.

Maspin has been characterized as a class II tumor suppressor based on its ability to inhibit cell invasion and angiogenesis and to promote apoptosis [48–50], suggesting maspin as a potential therapeutic target in multiple cancer types [51]. Several lines of evidence indicate the involvement of IKKα in regulating maspin expression. Downregulation of IKKα increased maspin expression to inhibit metastasis and promote apoptosis in HCC cells [30]. In addition, cytokine-activated nuclear IKKα repressed maspin transcription to control prostate cancer metastasis [34]. However, the detailed regulatory mechanism of IKKα-mediated maspin suppression remains unclear. Restoration of maspin expression by 5-aza-dc/TSA can synergistically enhance myocardin–induced apoptosis in MCF-7 cells, suggesting the involvement of promoter hypermethylation and histone hypoacetylation in maspin gene silence [52–54]. Although nuclear cytokine-activated IKKα was reported to repress maspin through DNA methylation in prostate cancer metastasis, the DNA demethylation drug 5-aza-2′-deoxycytidine didn’t restore the maspin expression in Hep3Bx cell (our unpublished data). Additionally, the maspin-3′UTR activity and maspin mRNA stability were significantly disrupted by nuclear IKKα, indicating that nuclear IKKα mainly suppressed maspin expression through post-transcriptional regulation in HCC. Our study provided a clear mechanism that HBx activated IKKα to nuclear translocation, and then phosphorylated H3 at Ser-10 to facilitate NFκB-mediated miRNA transcriptions to target maspin mRNA. Suppression of maspin by HBx-IKKα-NFκB-miRNA axis played a crucial role in HCC progression and chemoresistance. Blockade of nuclear IKKα function by silencing IKKα and inhibiting its nuclear translocation significantly retarded these microRNAs and their host gene expressions, and restored maspin protein expression and re-sensitized HBx-expressing HCC cells to doxorubicin. These data raised the possibility that combination treatment with IKKα specific inhibitor to restore maspin expression may improve the chemotherapeutic responses in HBV-associated HCC patients.

Recent studies have shown that microRNAs play essential roles in tumorigenesis, metastasis, and chemoresistance through the post-transcriptional regulation of tumor associated-genes [55–57]. MicroRNA-7 has been proposed controversially to function as an oncogene or a tumor-suppressor in different cancer types. Upregulation of microRNA-7 was observed in renal cell carcinoma and plays an important role in migration, cell proliferation, and apoptosis [58]; however, microRNA-7 inhibited metastasis and invasion through targeting focal adhesion kinase in cervical cancer [59]. In our previous study, induction of microRNA-7 by trichostatin A (TSA) suppressed the off-target effect of lapatinib on EGFR up-regulation, thus overcome the metastatic ability of HER2-negative breast cancer cells [60–62]. Although HBx-induced microRNA-7 downregulated EGFR expression to render HCC cells a slow-growth behavior [63], the expression of microRNA-7 was demonstrated regulated by nuclear IKKα and served as a oncogene to promote metastasis and chemoresistance through suppressing maspin expression in this study. Additionally, resveratrol increased the expressions of tumor suppressors, PDCD4 and maspin, to reduce prostate cancer growth and metastasis by inhibiting the Akt/microRNA-21 pathway [64]. These studies are consistent with our finding that HBx-activated nuclear IKKα transcriptionally upregulated maspin-targeting microRNA-7/-21/-103/-107 expression in HCC tumor progression. MicroRNA-103/107 have been identified as a invasive predictor of tumor relapse and overall survival for triple-negative breast cancer patients [65]; additionally, microRNA-103/-107 also modulated multiple drug resistance in human gastric carcinoma by downregulating caveolin-1 [66]. In contrast, microRNA-103/107 overexpression was considered as a possible chemosensitizer to promote genomic instability [67]. Interestingly, in high metastatic breast cancer cells, overexpression of microRNA-103/107 targeted and degraded NF-κB-interacting LncRNA, NKILA, which can interact with NF-κB/IκB to prevent overactivation of NF-κB pathway [68]. According to these studies, microRNA-103 and 107, which were transcriptionally upregulated by HBx-activated nuclear IKKα, might also negatively regulate the NKILA expression, thus provided positive feedback for enforcing NF-κB activation to suppress maspin expression and promote tumor progression in HCC cells.

**Figure 7:**
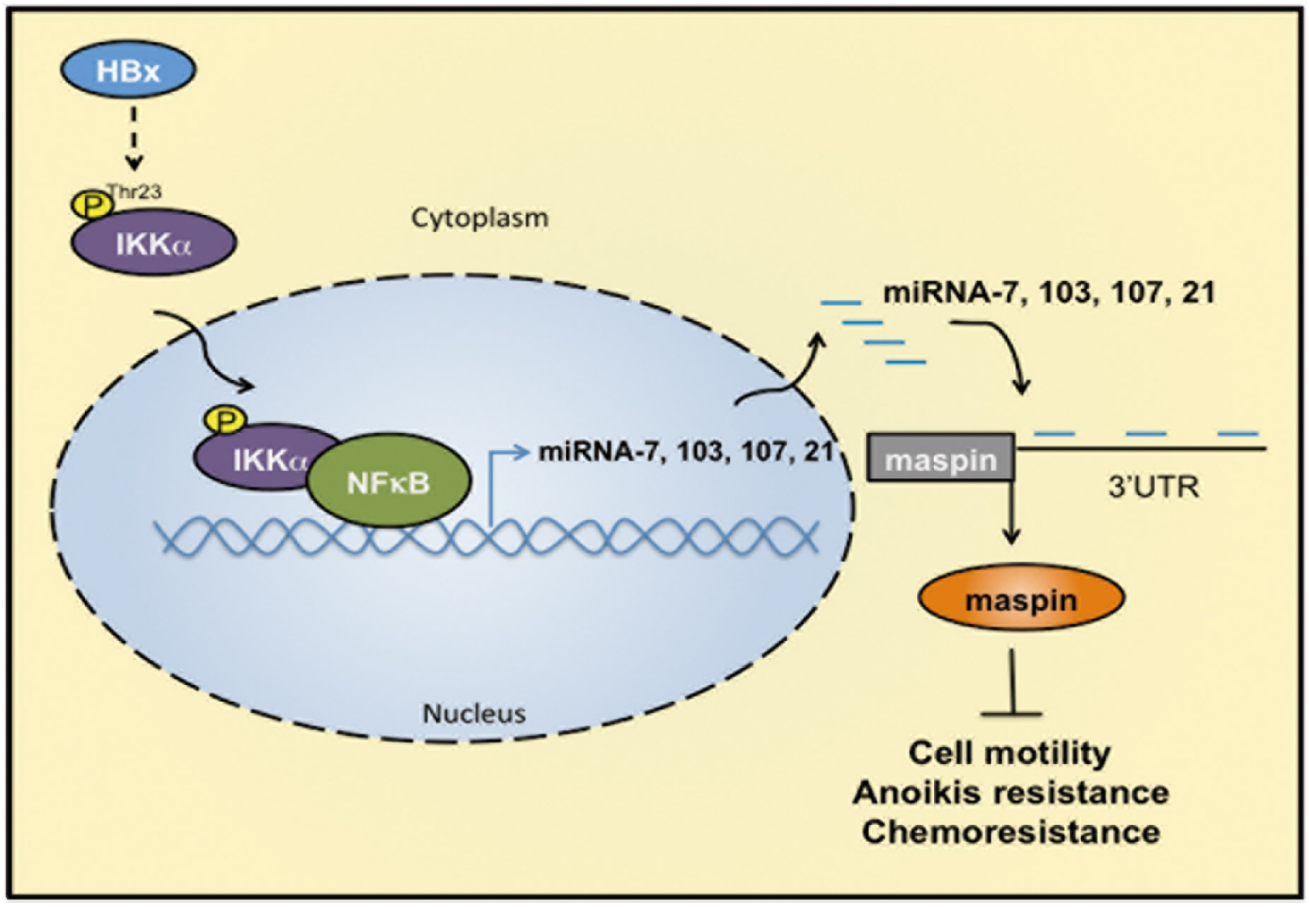
IKKα/NFκB-dependent microRNAs contribute to HBx-mediated hepatocellular tumor progression through suppression of maspin HBx induces IKKα phosphorylation at Thr-23, leading to its nuclear translocation. Nuclear IKKα cooperated with NFκB to induce miRNA-7, −103, −107, and −21 transcriptions for maspin downregulation by targeting its 3′UTR. Suppression of maspin leads to the motility, anoikis resistance, and chemoresistance of HCC cells.

Taken together, our study demonstrated that HBx transcriptionally enhanced the levels of microRNA-7, −103, −107, and −21 in a nuclear IKKα/NF-κB- manner. These elevated miRNAs directly targeted and suppressed maspin expression to promote HCC tumor progression and were strongly associated with the poor survival of HBV-related HCC patients (Figure 7). This study not only provides the molecular insight into the nuclear IKKα-mediated maspin suppression in response to HBx, but also highlights the possibility of IKKα-targeting therapy in the treatment of HCC patients.

## MATERIALS AND METHODS

### Cell culture

HEK-293, Hep3B, and Hep3Bx cell lines were cultured in Dulbecco's modified Eagle's medium/F12 medium supplemented with 10% fetal bovine serum.

### Plasmids, antibodies, and chemicals

Maspin-3′UTR was constructed in to pMIR-REPORT™ Luciferase plasmid (Ambion, Austin, TX, USA) [28]. Mutations of indicated sites in maspin-3′UTR and Flag-IKKα were generated using a Quickchange site-directed mutagenesis kit (Stratagene, La Jolla, CA, USA) according to the manufacturer's instructions. All of the above plasmids were confirmed by DNA sequencing. We purchased antibodies against IKKα, phospho-IKKα Thr23, IKKβ, and maspin from Santa Cruz (Santa Cruz, CA, USA), antibody against Flag-tag from Sigma-Aldrich (St. Louis, MO, USA), and antibody against phospho-IKKα Thr-23 (for IHC) from Abnova, The validated miRNA inhibitors were purchased from Dharmacon (Lafayette, CO, USA). The validated shRNA for negative control and IKKα were purchased from National RNAi Core Facility at Academia Sinica (Taipei, Taiwan). Tripure isolation reagent for RNA isolation was purchased from Roche (Indianapolis, IN, USA). The MMLV First-Strand cDNA Synthesis kit and Universal probelibrary Probe#21 was purchased from Roche (Indianapolis, IN, USA). The KAPA SYBR^®^ FAST Master Mix (2X) and KAPA Probe FAST Universal qPCR Kit were purchased from Kapa biosystem (Woburn, MA). The chemotherapeutic drugs, doxorubicin hydrochloride was purchased from Sigma-Aldrich (St. Louis, MO, USA). Luciferase assay system was purchased from Promega (Madison, WI, USA). Actinomycin D was purchased from Sigma-Aldrich (St. Louis, MO).

### Clinical specimens

HCC tissue sections and specimens were purchased from Taiwan Liver Cancer Network, Zhunan, Taiwan and provided from National Cheng Kung University Hospital, Tainan, Taiwan. Informed consents were signed by patients with approval by the Institutional Review Board, China Medical University Hospital, Taichung, Taiwan (DMR101-IRB1-119) and by the Institutional Review Board of the Human Investigation Committee of College of Medicine, National Cheng Kung University Tainan, Taiwan (B-ER-102-210). The clinical sample information met REMARK (REporting recommendations for tumor MARKer prognostic studies) guideline was shown in our previous study [28]

### Immunohistochemical Staining (IHC)

Five-micron thick paraffin-embedded tissue sections were deparaffinized and rehydrated. After antigen retrieval, the tissue sections were treated with Peroxidase Block, Protein block, and subsequently incubated with rabbit monoclonal anti-human maspin and anti-phospho-IKKα (Thr23) antibodies (100 dilution, Santa Cruz and Abnova, respectively) at 4°C for overnight. After washing to remove unbound primary antibody, sections were treated with a NovoLink Polymer anti-mouse/rabbit IgG-Poly-HRP according to manufacturer's instructions (NovoLinkTM polymer detection system, Leica) for 30 minutes. Tissue sections were incubated in the chromogenic peroxidase substrate, diaminobenzidine (DAB), for 30 second or 5 minutes, and subsequently counterstain with Hematoxylin for nucleus staining. The specificity of labeling by this procedure was verified by negative control reactions using buffer to replace the primary antibody and isotype-specific IgG.

### 3-(4,5-dimethylthiazol-2-yl)-2,5-diphenyl-tetrazolium bromide (MTT) cell viability assay

*In vitro* cell viability was measured using an MTT colorimetric assay. Hep3Bx cells (1×10^4^ cells/well) were seed in 96-well plate. After IKK inhibitor VII treatment, the culture medium was removed and 1μg/ml MTT solution (Sigma, St. Louis, MO, USA) was added to incubate for 3 hours. Finally, DMSO was added to lyse the cells and the absorbance at OD_550_ wavelength was detected by ELISA reader.

### Reporter gene luciferase assay

As described previously [69], cells with 60–80% of confluence were transfected with maspin-3′UTR luciferase plasmids and maspin promoter plasmid along with or without miRNA inhibitors, myc-HBx, or IKKα expression vectors. After 48hrs of transfection, cell lysates were harvested and subjected to luciferase assay system. Luciferase activity was normalized to β-gal activity.

### Preparation and infection of shRNA-IKKα expressing lentivirus

Briefly, 2 μg pCMV-dR8.91, 200 μg pMD2.G, and 2 μg pLKO-shLuciferase, or pLKO-shIKKα were cotransfected into HEK293T cells using Lipofectamine 2000. The supernatants containing infectious lentivirus were collected after 1 day of transfection. For lentivirus infection, cells (2×10^5^) were infected with lentivirus at a multiplicity of infection (MOI). After 5 days infection, cells were harvested for protein or RNA extraction.

### Quantitative real-time PCR (RT-qPCR)

Total RNA was extracted by using Tripure isolation reagent according previous report [70]. One μg of RNA was subjected to reverse transcription with the MMLV First-Strand cDNA Synthesis kit. After reverse transcription, the qPCR analysis of maspin, IKKα, PGSF1, PANK2, and PANK1 mRNA expressions was performed on ABI 7500 system (Applied Biosystems, Foster, CA) by using KAPA SYBR^®^ FAST Master Mix (2X) and was normalized to GAPDH expression. The qPCR analysis of miR-7, −103, −107, and −21 expression was performed on LightCycler 480 System (Roche, Indianapolis, IN, USA) by using KAPA Probe FAST Universal qPCR Kit and was normalized to U48 expression. Specific primers used in real-time PCR were listed in Supplementary Table.

### Chromatin immunoprecipitation assay

Cells were cross-linked with 1.42% formaldehyde for 15 min and quenched unreacted formaldehyde with 0.125 M Glycine solution for 5 min. Cells were scraped in 1 ml of cold PBS, centrifuged, and lysed in 1mL of IP buffer (150mM NaCl, 50mM Tris-HCl, pH 7.5, 5mM EDTA, 0.5% Nonidet P-40, and 1% Triton X-100) containing protease inhibitors (1mM phenylmethylsulfonyl fluoride, 1μM eupeptin and 1μM aprotinin). The nuclear pellet was resuspended in IP buffer and sonicated to shear chromatin. The sonicated lysates were immunoprecipited with antibodies against phospho-H3^ser10^ followed by the pull-down with protein A/G-Sepharose (Thermo). The immunoprecipitated DNA and input DNA were extracted by incubating with 100 μl of 10% Chelex (Bio-Rad), boiling to reverse the cross-link, and centrifuging to remove Chelex slurry. RT-qPCR was performed with the purified DNA using specific primers as shown in Supplementary Table.

### Transient transfection

Cells at 60% confluence were transfected with indicated plasmids or microRNA inhibitors using Nanofectin (PAA, Pasching, Austria), Lipofetamine 2000 (Invitrogen, Boston, USA), or DharmaFECT (Thermo Scientific Dharmacon, Lafayette, CO, USA) as described previously [71]. Nanofectin and Lipofectamine 2000 were used in plasmid DNA transfection, and the DharmaFECT was used in microRNA inhibitor transfection. After transfection for 48 h, cells were subjected to total lysate preparation, total RNA extraction, or luciferase assays.

### Protein extraction and immunoblot

For total cell lysates, cells were washed with ice-cold PBS one time and lysed in RIPA buffer (20 mM Tris-HCl, pH7.4, 150 mM NaCl, 1% NP-40, 1% sodium deoxycholate, 1 mM EDTA and 1 mM EGTA) containing protease inhibitors and phosphatase inhibitors cocktails (Roche, Indianapolis, IN, USA). Proteins were separated by SDS-PAGE, transferred to PVDF membrane, and blotted with indicated antibodies.

### Statistical analysis

The difference in relative gene expression between tumor and normal tissues was calculated by a two-tailed Student's t test. Coefficient analyses were performed for the correlation between gene expressions. The percentage of cumulative survival was determined by Kaplan-Meier survival test. The univariate and multivariate analyses were used in Cox proportional hazards models. All these statistical analyses were performed using Sigma Plot 10.0. A p-value < 0.05 was defined as statistically significant.

## SUPPLEMENTARY MATERIAL TABLES



## Abbreviations

Maspin: Mammary serine protease inhibitor
HCC: hepatocellular carcinoma
IKKα: kinase alpha
miR: microRNA
HBV: hepatitis virus B
HBx: HBV X protein
NF-κB: nuclear factor kappa B
UTR: untranslational region
NLS: nuclear localization signal
NES: nuclear export signal
shRNA: small-hairpin RNA
CBP: CREB-binding protein
PGSF: pituitary gland specific factor
PANK: pantothenate kinase.
