# Supplementary material for: Nuclear IKKα mediates microRNA-7/-103/107/21 inductions to downregulate maspin expression in response to HBx overexpression
Source: Oncotarget. 2016 Jul 7;7(35):56309–23. doi: 10.18632/oncotarget.10462 (PMC5302916; doi:10.18632/oncotarget.10462)
Supplement: Supplementary file 1 [file oncotarget-07-56309-s001.pdf]

Nuclear IKKα mediates microRNA-7/-103/107/21 inductions to downregualte maspin expression in response to HBx overexpression

SUPPLEMENTARY TABLE

Supplementary Table S1: Primers used in this study

RT-qPCR primer	Sequence (5′-3′)
maspin	Forward CATGTTCATCCTACTACCCAAGG<br>Reverse TCTGAGTTGAGTTGTTTTTCAATCTT
IKKα	Forward ACCATTTGCATCCAGAAGTTTATC<br>Reverse TGCTCAGGTGACCAAACAGCT
PGSF1	Forward CTTGGGAGCACCAAATATC<br>Reverse AATAAGGATCGTTTCCCTTG
PANK2	Forward AGAGGAAGAGGTGGAAAGTC<br>Reverse GTGGGAAAGCGTATAAAGTG
PANK1	Forward CTTTGGCAACATGATGAGTA<br>Reverse GAGCTTTCAGTTGTCCTTTG
GAPDH	Forward AGCCACATCGCTCAGACAC<br>Reverse GCCCAATACGACCAAATCC
miR-7	Forward GTTGGCTCTGGTGCAGGGTCCG<br>AGGTATTCGCACCAGAGCCAACACAACA<br>Reverse GCGGCGTGGAAGACTAGTGAT
miR-103	Forward GTTGGCTCTGGTGCAGGGTCCGAG<br>GTATTCGCACCAGAGCCAACTCATAG<br>Reverse GCGTCCAGCAGCATTGTACAG
miR-107	Forward GTTGGCTCTGGTGCAGGGTCC<br>GAGGTATTCGCACCAGAGCCAACTGATAG<br>Reverse GGTGGCAGCAGCATTGTACAG
miR-21	Forward GTTGGCTCTGGTGCAGGGTCCGA<br>GGTATTCGCACCAGAGCCCAACTCAACA<br>Reverse GGCGGCTAGCTTATCAGACTG
snRNA U48	Forward GTTGGCTCTGGTGCAGGGTCCG<br>AGGTATTCGCACCAGAGCCAACTCAGCG<br>Reverse CGGCGGTAACTCTGAGTGTGT
snRNA U6B	Forward GTTGGCTCTGGTGCAGGGTCCGA<br>GGTATTCGCACCAGAGCCAACAAAAATAT<br>Reverse TTCCTCCGCAAGGATGACACGC
Universal reverse primer #21	GTGCAGGGTCCGAGGT
ChIP qPCR primers	Sequence (5′-3′)
miR-7-3 promoter	Forward CTGGGACCCAGCAGAGGAAT<br>Reverse TGGTCTATTCCCTTGGCCCC
miR-103-2 promoter	Forward GACTCTGCCTGGTGATAGAC<br>Reverse GTCTCAAACTCCTGGCTTC
miR-107 promoter	Forward GCGTGCCAGCAAAGAAATTT<br>Reverse TCCCCAGTGAGCAAGATACC
miR-21 promoter	Forward CTTCTGACTAGTGGGAGGTG<br>Reverse ATGCTTGTGTCATCCCTAGT
